# Drug exposure in a metastatic human lung adenocarcinoma cell line gives rise to cells with differing adhesion, proliferation, and gene expression: Implications for cancer chemotherapy

**DOI:** 10.3892/mmr.2015.3837

**Published:** 2015-05-25

**Authors:** HUILING LI, JIANXING HE, NANSHAN ZHONG, ROBERT M HOFFMAN

**Affiliations:** 1 Department of Respiratory, Hainan Branch of PLA Hospital, Sanya, Hainan 572000, P.R. China; 2 Guangzhou Institute of Respiratory Disease, State Key Laboratory of Respiratory Disease, The First Affiliated Hospital of Guangzhou Medical University, Guangzhou, Guangdong 510120, P.R. China; 3 Department of Surgery, University of California, San Diego, CA 92111, USA; 4 AntiCancer, Inc., San Diego, CA 92111, USA

**Keywords:** lung adenocarcinoma cell line, drug resistance, cell adhesion, cell proliferation, green fluorescent protein, red fluorescent protein

## Abstract

The Am1010 cell line was previously established from a metastatic deposit in an arm muscle from a patient with lung adenocarcinoma who had undergone four cycles of chemotherapy with cisplatin and taxol. Am1010 cells were labeled with red fluorescent protein or green fluorescent protein. A total of eight sublines were isolated following *in vitro* exposure to cisplatin or taxol. The sublines differed with regard to their adhesion and proliferation properties, with certain sublines exhibiting an increased proliferation rate and/or decreased surface adhesion. Gene expression assays demonstrated that tenascin C; cyclin D1; collagen, type 1, α2; integrin α1; related RAS viral (r-ras) oncogene homolog 2; platelet-derived growth factor C; and Src homolog 2 domain containing in the focal adhesion pathway, and intercellular adhesion molecule 1, F11 receptor, claudin 7 and cadherin 1 in the cell adhesion pathway, varied in expression among the sublines. The results of the present study suggested that drug exposure may alter the aggressiveness and metastatic potential of cancer cells, which has important implications for cancer chemotherapy.

## Introduction

Lung cancer is the leading cause of mortality among all types of malignancy (1). Regardless of the treatment provided, the 5-year survival rate in lung cancer is <15%. The poor prognosis is predominantly attributed to the development of drug resistance (2). It is therefore important to identify the mechanisms underlying drug resistance in lung cancer.

Cancer stem cells (CSCs) exhibit increased drug resistance and tumorigenicity (3,4). Levina *et al* (1) suggested that CSCs may be enriched and subsequently isolated from cancer cell populations following drug treatment. The authors isolated what they termed drug-surviving cells (DSCs) from human cancer cell lines treated with cisplatin, doxorubicin or etoposide. The isolated DSCs were clonogenic, expressed CSC cell surface and embryonic stem cell markers, exhibited self-renewal and differentiation, and were tumorigenic and metastatic in severe combined immunodeficiency mice. It was concluded that the DSCs were CSCs and that enrichment of CSCs following drug treatment *in vitro* may result in a similar selection of drug-resistant CSCs in patients during chemotherapy (1).

Our group previously established the cell line Am1010 (5) directly from a lung cancer patient who was treated with chemotherapy but developed multidrug resistance. In the present study, the establishment of eight sublines of DSCs from Am1010, labeled with red fluorescent protein (RFP) or green fluorescent protein (GFP), by long-term exposure to cisplatin or taxol is described. Cell proliferation and gene expression were then determined, in order to define the differences between the sublines.

## Materials and methods

### Ethics statement

All experimentation presented in the current study has been approved by the local institutional review board. The tumor sample was obtained from the Department of Thoracic Surgery at the 1st Affiliated Hospital of Guangzhou Medical College with the approval of the local ethical committee. Written informed consent was obtained from the patient.

### RFP or GFP expression in Am1010 cells

The RFP (DsRed-2) gene (Clontech Laboratories, Mountain View, CA, USA) was inserted in the retroviral-based mammalian expression vector, pLNCX (Clontech Laboratories), to form the pLNCX DsRed-2 vector. The EGFP gene (Clontech Laboratories) was inserted into the retroviral-based mammalian expression vector, pLEIN, to form the pLEIN EGFP vector. Transfection of pLNCX DsRed-2 or pLEIN GFP into PT67 packaging cells produced retroviral supernatants containing the *DSRed-2* or *EGFP* gene. Briefly, PT67 cells were grown as monolayers in Dulbecco's modified Eagle's medium supplemented with 10% fetal bovine serum (FBS; Gemini Biological Products, Calabasas, CA, USA). Exponentially growing cells in 10-cm dishes were transfected with 10 *µ*g of the expression vector using Lipofectamine^®^ and Plus reagent (Invitrogen Life Technologies, Carlsbad, CA, USA). Transfected cells were replated 48 h after transfection and 100 *µ*g/ml G418 was added. After two days, the quantity of G418 was increased to 200 *µ*g/ml. During the drug selection period, surviving colonies were visualized under fluorescence microscopy and RFP-positive and GFP-positive colonies were isolated (6).

For RFP or GFP gene transduction, cells were incubated at 20% confluence with a 1:1 precipitated mixture of retrovirus-containing supernatants of PT67 cells and RPMI 1640 or other culture medium (Invitrogen Life Technologies) containing 10% FBS (Gemini Biological Products) for 72 h. Cells were replenished with fresh medium at this time. Cancer cells were harvested with trypsin/EDTA and subcultured at a ratio of 1:15 into selective medium, which contained 50 *µ*g/ml G418. In order to select brightly fluorescent cells, the concentration of G418 was increased to 800 *µ*g/ml in a stepwise manner. The clones of cancer cells expressing RFP or GFP were isolated using cloning cylinders (Bel-Art Products, Wayne, NJ, USA) by trypsin/EDTA and amplified and transferred by conventional culture methods in the absence of the selective agent (6).

### Establishment of DSC sublines

Once Am1010 cells (5) had grown to 80% confluence, cisplatin (1 *µ*M) was added to the medium for 1 month. Suspended cells appeared following drug exposure and were transferred to a new culture dish. Am1010 cells were also exposed to taxol (0.1 *µ*M) in a procedure similar to that used for cisplatin. Am1010-cis-suspension-GFP, Am1010-cis-adhesion-GFP, Am1010-tax-suspension-GFP, Am1010-tax-adhesion-GFP, Am1010-cis-suspension-RFP, Am1010-cis-adhesion-RFP, Am1010-tax-suspension-RFP and Am1010-tax-adhesion-RFP cells were isolated by culture with cisplatin or taxol. When the DSC sublines were passaged, the suspended or attached status of the subline was maintained for 30–100 h. After this time period, the suspended cells attached and proliferated. The cells were subsequently cultured as normal cells, and their gene expression of CCND1, TNC, COL1A2, ITGA1, RRAS2, PDGFC, SHC1, ICAM1, CLDN7, F11R and CDH1 was assayed every three months, in order to observe their stability. Unstable cells were discarded.

### Reverse transcription-quantitative polymerase chain reaction (RT-qPCR)

The expression of tenascin C (TNC); cyclin D1 (CCND1); collagen, type 1, α2 (COL1A2); integrin α1 (ITGA1); related RAS viral (r-ras) oncogene homolog 2 (RRAS2); platelet-derived growth factor C (PDGFC); Src homolog 2 domain containing (SHC1); intercellular adhesion molecule 1 (ICAM1); F11 receptor (F11R); claudin 7 (CLDN7); and cadherin 1 (CDH1) was analyzed by SYBR Green qPCR. Total RNA was isolated from cultured cells, omental tissues and oral mucosal tissues using the TRIzol^®^ method (Invitrogen Life Technologies). The total quantity of RNA was determined using a Nanodrop spectrophotometer (ND1000; NanoDrop Technologies, Wilmington, DE, USA). The purity was assessed using denaturing agarose gel electrophoresis. cDNA synthesis was conducted with 1 mg of RNA using the High Capacity cDNA Reverse Transcription kit (Invitrogen Life Technologies). RT-qPCR was performed using an ABI Prism 7900HT system (Applied Biosystems, Foster City, CA, USA). RT-qPCR reactions were conducted using 26 Power SYBR Green PCR master mixes (Applied Biosystems) according to the manufacturer's instructions. For PCR amplification, an initial step at 50°C for 2 min was performed, followed by a denaturation step at 95°C for 15 min. Subsequently, 45 cycles of a denaturation step (95°C for 15 sec) and an annealing and extension step (60°C for 60 sec) were performed. PCR reactions were performed in triplicate for each sample. A dissociation reaction was also conducted for each primer in order to assess its specificity. The relative quantification (ΔΔCt method), which describes the change in expression of the target gene in a test sample relative to that in a calibrator sample, was used to analyze the data. Data were analyzed using the 7900HT sequence detector system software version 2.2.1 (Applied Biosystems). Results were expressed relative to the expression levels of internal reference genes (β-actin and GAPDH). Details of the primers used for specific genes are presented in Table I.

### Cell proliferation measurements

Aliquots (100 *µ*l) of exponentially growing cell suspensions (5×10^4^ cells/ml) were seeded in 96-well microtiter plates and incubated for 24 h. At 0, 24, 48 and 96 h, 20 *µ*l of 3-(4,5-dimethylthiazol-2-yl)-2,5-diphenyltetrazolium bromide solution (5 mg/ml in phosphate-buffered saline) was added to each well and the plates were incubated at 37°C for an additional 3 h. Following centrifugation of the plates at 800 × g for 5 min, the medium was aspirated from each well as fully as possible and 200 *µ*l of dimethyl sulfoxide was added to each well to dissolve the formazan crystals. The optical density was measured at 490 nm using the Delta-soft ELISA analysis software interfaced to a Bio-Tek microplate reader (EL-340; Biometallics, Inc., Princeton, NJ, USA).

### Statistical analysis

Differences in proliferation between different cell lines were analyzed using Student's t-test. Statistical analysis was performed using SPSS 13.0 softward (SPSS, Inc., Chicago, IL, USA). P<0.05 was considered to indicate a statistically significant difference.

## Results

### Labeling of Am1010 sublines with GFP and RFP

**Figure 1:**
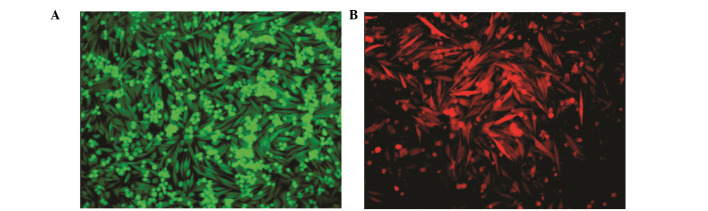
Am1010 fluorescent cells. (A) Am1010-GFP. The EGFP was expressed in Am1010 cells. (B) Am1010-RFP. The RFP (dsRed-2) gene was expressed in Am1010 cells. Magnification, ×100. GFP, green fluorescent protein; RFP, red fluorescent protein.

Am1010 cells were stably labeled with GFP or RFP. Fig. 1 shows Am1010-GFP and Am1010-RFP cells. GFP and RFP expression did not alter the proliferation rate of the cells (data not shown).

### DSC sublines of Am1010-GFP or Am1010-RFP had differing adhesion properties

**Figure 2:**
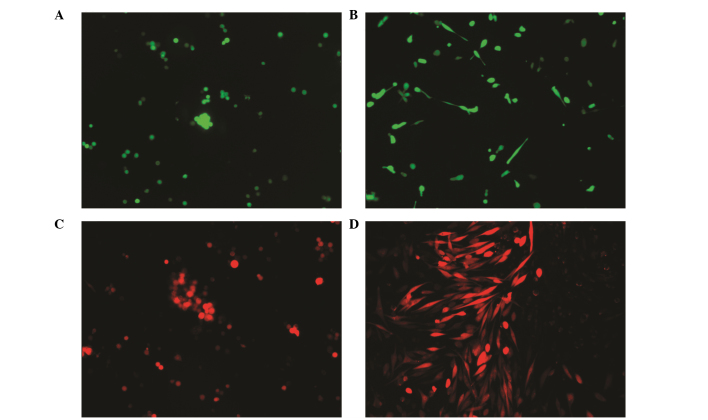
Am1010 sublines isolated by cisplatin exposure. (A) Am1010-cis-suspension-GFP: Suspended subline isolated by cisplatin exposure of Am1010-GFP. (B) Am1010-cis-adhesion-GFP: Attached subline isolated by cisplatin exposure of Am1010-GFP. (C) Am1010-cis-suspension-RFP: Suspended subline isolated by cisplatin exposure of Am1010-RFP. (D) Am1010-cis-adhesion-RFP: Attached subline isolated by cisplatin exposure of Am1010-RFP. Magnification, ×100. GFP, green fluorescent protein; RFP, red fluorescent protein.

**Figure 3:**
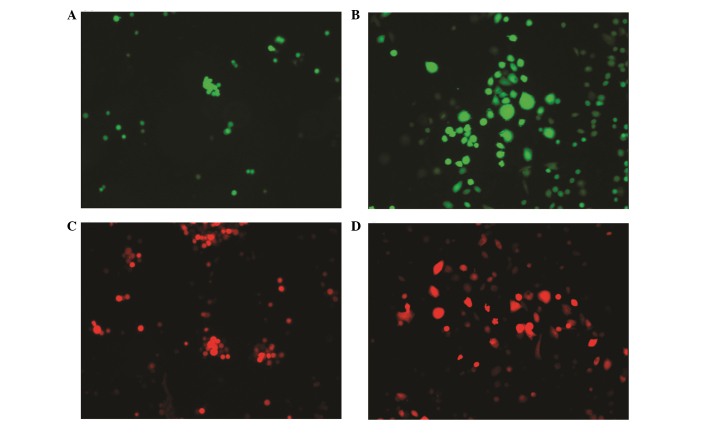
Am1010 sublines isolated by taxol exposure. (A) Am1010-tax-suspension-GFP: Suspended subline isolated by taxol exposure of Am1010-GFP. (B) Am1010-tax-adhesion-GFP: Attached subline isolated by taxol exposure of Am1010-GFP. (C) Am1010-tax-suspension-RFP: Suspended subline isolated by taxol exposure of Am1010-RFP. (D) Am1010-tax-adhesion-RFP: Attached subline isolated by taxol exposure of Am1010-RFP. Magnification, ×100. GFP, green fluorescent protein; RFP, red fluorescent protein.

Exposure of Am1010-GFP or Am1010-RFP cells to cisplatin or taxol, enabled the isolation of suspended or attached sublines. The following sublines were selected: Am1010-cis-suspension-GFP, Am1010-cis-adhesion-GFP, Am1010-cis-suspension-RFP, Am1010-cis-adhesion-RFP (Fig. 2), Am1010-tax-suspension-GFP, Am1010-tax-adhesion-GFP, Am1010-tax-suspension-RFP and Am1010-tax-adhesion-RFP (Fig. 3). When the DSC sublines were passaged, the suspension or attached status of the subline was maintained for 30–100 h. After this time period, the suspended cells attached and proliferated.

### Gene expression of TNC, CCND1, COL1A2, ITGA1, RRAS2, PDGFC, SHC1, ICAM1, F11R, CLDN7 and CDH1 in the DSC sublines, Am1010-GFP and Am1010-RFP

**Figure 4:**
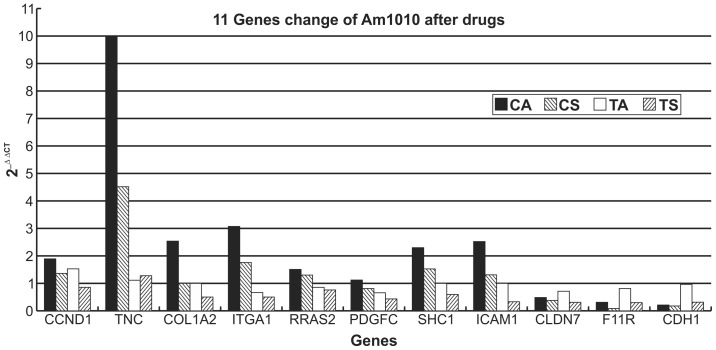
Relative gene expression changes in different sub-cell lines. Am1010 cells that attached poorly following drug exposure demonstrated upregulation of CCND1, TNC, COL1A2, ITGA1, RRAS2, PDGFC and SHC1 expression in the focal-adhesion pathway, and ICAM1 expression in the cell-adhesion pathway, in addition to downregulation of F11R, CLDN7 and CDH1 expression in the cell-adhesion pathway. Cells with improved attachment following drug exposure demonstrated that all eleven genes exhibited slight changes in the expression levels in cells with improved attachment. CCND1, cyclin D1; TNC, tenascin C; COL1A2, collagen, type 1, α2; ITGA1, integrin α1; RRAS2, related RAS viral (r-ras) oncogene homolog 2; PDGFC, platelet-derived growth factor C; SHC1, Src homolog 2 domain containing; ICAM1, intercellular adhesion molecule 1; CLDN7, claudin 7; F11R, F11 receptor; CDH1, cadherin 1; CA, Am1010-cis-adhesion; CS, Am1010-cis-suspension; TA; Am1010-tax-adhesion; TS, Am1010-tax-suspension; cis, cisplatin; tax, taxol.

Am1010-cis-adhesion and Am1010-cis-suspension cells exhibited the same trend for the expression of the eleven genes measured. Expression of TNC, CCND1, COL1A2, ITGA1, RRAS2, SHC1 and ICAM1 was upregulated and that of F11R, CLDN7 and CDH1 expression was downregulated (all P<0.01), as compared with the Am1010 cells which have a basic value of 1 in Table II and Fig. 4, and the Am1010-tax-adhesion and Am1010-tax-suspension cells (all P<0.01). PDGFC expression exhibited little variation, as compared with the Am1010-tax-adhesion and Am1010-tax-suspension cells (P>0.05). Am1010-cis-adhesion cells revealed a greater degree of upregulation of gene expression than Am1010-cis-suspension cells. By contrast, Am1010-cis-suspension cells demonstrated a greater degree of downregulation of gene expression than Am1010-cis-adhesion cells.

All eleven genes exhibited little variation of expression in Am1010-tax-adhesion cells (P>0.05). By contrast, the expression of almost all genes, with the exception of TNC, which exhibited little variation, was downregulated in Am1010-tax-suspension cells (P<0.05; Table II and Fig. 4).

### Differential cell proliferation rates in DSC sublines derived from Am1010-GFP and Am1010-RFP cells

**Figure 5:**
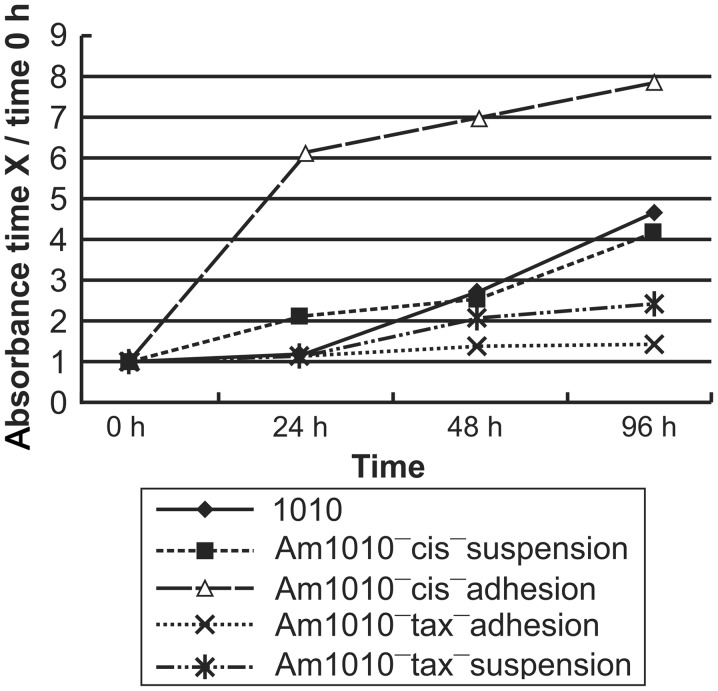
Proliferation rates of the sublines. Am1010-cis-suspension, Am1010-cis-adhesion, Am1010-tax-suspension, and Am1010-tax-adhesion. Cis, cisplatin; tax, taxol.

The cell proliferation rate differed among the DSC sublines derived from Am1010-GFP and Am1010-RFP cells (P>0.05; Fig. 5). Am1010-cis-suspension-GFP cells and Am1010-cis-suspension-RFP cells were thus termed Am1010-cis-suspension cells. Am1010-cis-adhesion-GFP cells and Am1010-cis-adhesion-RFP cells were termed Am1010-cis-adhesion cells. Am1010-tax-suspension-GFP cells and Am1010-tax-suspension-RFP cells were termed Am1010-tax-suspension cells. Am1010-tax-adhesion-GFP cells and Am1010-tax-adhesion-RFP cells were termed Am1010-tax-adhesion cells. Am1010-cis-adhesion cells and Am1010-tax-adhesion cells had the highest and lowest proliferation rates, respectively (P<0.01).

## Discussion

The present study describes the establishment of eight DSC sublines expressing either GFP or RFP, derived by drug exposure from the Am1010 cell line (5), which was established from a metastasis resected from a patient with lung cancer. These cell lines demonstrated *in vitro* multidrug resistance to cisplatin and taxol. Exposure of Am1010 cells *in vitro* to cisplatin or taxol resulted in sublines with varied proliferation and ability to attach to a cell culture dish.

The variability in the ability to attach to a cell culture dish indicated that the expression of certain genes associated with the adhesion pathway of Am1010 cells may vary following exposure to chemotherapy. In our previous study, eleven adhesion pathway genes, TNC, CCND1, COL1A2, ITGA1, RRAS2, PDGFC, SHC1, ICAM1, F11R, CLDN7 and CDH1 were observed to be differentially expressed in a microarray analysis comparing expression in Am1010 cells with that in P0318 cells (5). In contrast to Am1010 cells, P0318 is a non-drug-surviving cell line. The patient from whom this cell line was obtained had not undergone chemotherapy and exhibited the same pathology as that of the donor of the Am1010 cell, with the exception of the presence of metastases (5). The differential expression of these genes in the two cell lines may be associated with their differing metastatic ability. TNC, CCND1, COL1A2, ITGA1, RRAS2, PDGFC and SHC1 are genes involved in the focal adhesion pathway and ICAM1, F11R, CLDN7 and CDH1 are genes involved in the cell-adhesion pathway. The two pathways have important roles in cancer metastasis. The expression of these genes was consequently evaluated following drug exposure. The drug concentration of cisplatin and taxol in the cell cultures was 1 *µ*M and 0.1 *µ*M, respectively, which was similar to the levels in the body when cisplatin was used at the dose of 80–120 mg/m^2^ and taxol is used at the dose of 135–250 mg/m^2^.

Following cisplatin exposure, all cells exhibited a similar expression pattern for each of the eleven genes. TNC, CCND1, COL1A2, ITGA1, RRAS2, SHC1 and ICAM1 expression was upregulated and F11R, CLDN7 and CDH1 expression was downregulated. PDGFC expression exhibited little variation. Gene expression was more markedly upregulated in cells with improved attachment and more distinctly downregulated in cells with poorer attachment. Following taxol exposure, all eleven genes exhibited little change in expression in cells with improved attachment. By contrast, almost all of the evaluated genes were downregulated in cells that attached poorly, with the exception of TNC, which exhibited little change in expression. The differences in the adhesion properties of the sublines suggest that drug exposure may alter the aggressiveness and metastatic potential of cancer cells, which has important implications for cancer chemotherapy.

Cell proliferation assays indicated very different growth rates among the DSC sublines derived from Am1010-GFP cells or Am1010-RFP cells. Am1010-cis-adhesion-GFP cells grew at the fastest rate. Thus, drug resistance may led to an acceleration of the growth of cancer cells. Enhanced proliferation may make a tumor more aggressive. The results of the present study suggested that chemotherapy in patients with lung cancer may give rise to DSCs with altered proliferation and metastasis.

Levina *et al* (1) suggested that CSCs may be enriched and subsequently isolated from cancer cell populations following drug exposure. The authors isolated DSCs from human cancer cell lines treated with cisplatin, doxorubicin or etoposide, and concluded that the DSCs were CSCs. Levina *et al* (1) stated that enrichment of CSCs following drug treatment *in vitro* suggests that a similar positive selection of drug-resistant CSCs may occur in patients during chemotherapy.

Studies by these group have demonstrated that drug exposure of cancer cells may result in DSCs, which vary significantly in proliferation, adhesion and gene expression (1,7). The present data suggested that drug exposure in cancer cells may generate highly aggressive variants. These results have important implications for the chemotherapy of lung cancer.

## Figures and Tables

**Table I:** Gene expression analysis.

Gene	GB.accession	Primer sequence 5′→3′	Amplificon size (bp)
CCND1	NM_053056	Forward AGAACACGGCTCACGCTTAC	204
		Reverse CCCAGACCCTCAGACTTGC	
TNC	NM_002160	Forward GAGATGCCAAGACTCGCTACA	182
		Reverse GTTGACACGGTGACAGTTCCT	
COL1A2	NM_000089	Forward CTACCCAACTTGCCTTCATG	229
		Reverse GTCTTTCCCCATTCATTTGTC	
ITGA1	NM_181501	Forward TGGCTTCTGAATGAAATACGA	109
		Reverse TTCTTTGGGTCACATACTGGA	
RRAS2	NM_012250	Forward GTGGTAGAACTTTTACTTGCTGG	116
		Reverse AGTGATTTCAGAGTCTCATCCTG	
PDGFC	NM_016205	Forward GTTCTTTCGATACGGCTTAGG	126
		Reverse CCAGATTTTATACGATTTTAGGC	
SHC1	NM_003029	Forward CTATGTACTCTACGCCAAAGTGC	183
		Reverse TATGTGGGGATTGTCTACTGC	
ICAM1	NM_000201	Forward GACCCCAACCCTTGATGATA	266
		Reverse AGTGCTTTTGTGCCGATAGA	
CLDN7	NM_001307	Forward ATGTATAGTCCTCTTGGGTTGG	215
		Reverse TCAGTGGGGTGCTAAGTGTTC	
F11R	NM_016946	Forward TCATCTTGTAACTGAAAGCGTG	110
		Reverse CTAACTCCGTTTTCCTCCACTA	
CDH1	NM_004360	Forward GAGGATGATTGAGGTGGGTC	114
		Reverse GGGATTCTGGGCTTTGAGTA	
GAPDH	NM_002046	Forward TGTTGCCATCAATGACCCCTT	202
		Reverse CTCCACGACGTACTCAGCG	
β-actin	NM_001101	Forward CATGTACGTTGCTATCCAGGC	250
		Reverse CTCCTTAATGTCACGCACGAT	

CCND1, cyclin D1; TNC, tenascin C; COL1A2, collagen, type 1, α2; ITGA1, integrin α1; RRAS2, related RAS viral (r-ras) oncogene homolog 2; PDGFC, platelet-derived growth factor C; SHC1, Src homolog 2 domain containing; ICAM1, intercellular adhesion molecule 1; CLDN7, claudin 7; F11R, F11 receptor; CDH1, cadherin 1.

**Table II:** Relative gene expression changes in different cell sublines.

2^−ΔΔCT^	Am1010-cis-adhesion	Am1010-cis-suspension	Am1010-tax-adhesion	Am1010-tax-suspension
CCND1	1.92	1.34	1.53	0.88
TNC	9.99	4.53	1.11	1.25
COL1A2	2.55	1.01	1.00	0.51
ITGA1	3.06	1.78	0.70	0.48
RRAS2	1.50	1.31	0.85	0.75
PDGFC	1.11	0.81	0.66	0.47
SHC1	2.29	1.54	0.94	0.57
ICAM1	2.54	1.33	1.01	0.30
CLDN7	0.52	0.38	0.71	0.31
F11R	0.31	0.11	0.83	0.31
CDH1	0.21	0.19	0.93	0.34

CCND1, cyclin D1; TNC, tenascin C; COL1A2, collagen, type 1, α2; ITGA1, integrin α1; RRAS2, related RAS viral (r-ras) oncogen homolog 2; PDGFC, platelet-derived growth factor C; SHC1, Src homolog 2 domain containing; ICAM1, intercellular adhesion molecule 1; CLDN7, claudin 7; F11R, F11 receptor; CDH1, cadherin 1.

## Abbreviations

DSC: drug surviving cell
CSC: cancer stem cell
